# Supplementary material for: Additive effect of contrast and velocity suggests the role of strong excitatory drive in suppression of visual gamma response
Source: PLoS One. 2020 Feb 13;15(2):e0228937. doi: 10.1371/journal.pone.0228937 (PMC7018047; doi:10.1371/journal.pone.0228937)
Supplement: S1 Fig — Correlations between GR power values measured at different velocities in 100% and 50% contrast conditions. (PDF) [file pone.0228937.s001.pdf]

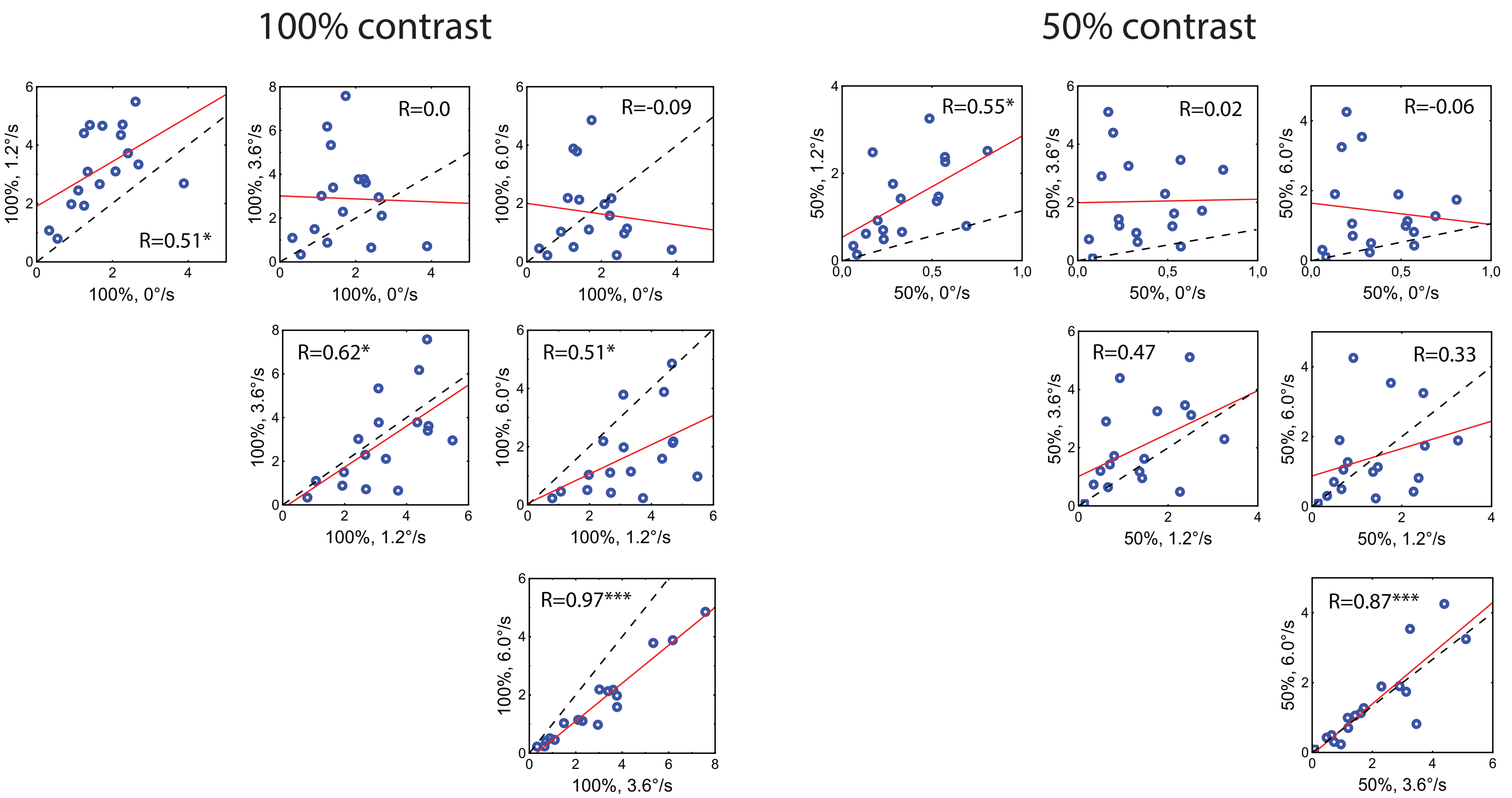

S1 Fig. Correlations between GR power values measured at different velocities in 100% and 50% contrast conditions. Blue dots denote individual gamma response power values measured as (Pow_{post}- Pow_{pre})/ Pow_{pre}. The linear regression is shown in red. The dashed line corresponds to the axis of symmetry.

Orekhova et al. PLOS One, 2020
