# Supplementary material for: Additive effect of contrast and velocity suggests the role of strong excitatory drive in suppression of visual gamma response
Source: PLoS One. 2020 Feb 13;15(2):e0228937. doi: 10.1371/journal.pone.0228937 (PMC7018047; doi:10.1371/journal.pone.0228937)
Supplement: S2 Fig — Distributions of individual peak frequencies of gamma responses induced by 50% and 100% contrast gratings moving at different velocities. (PDF) [file pone.0228937.s002.pdf]

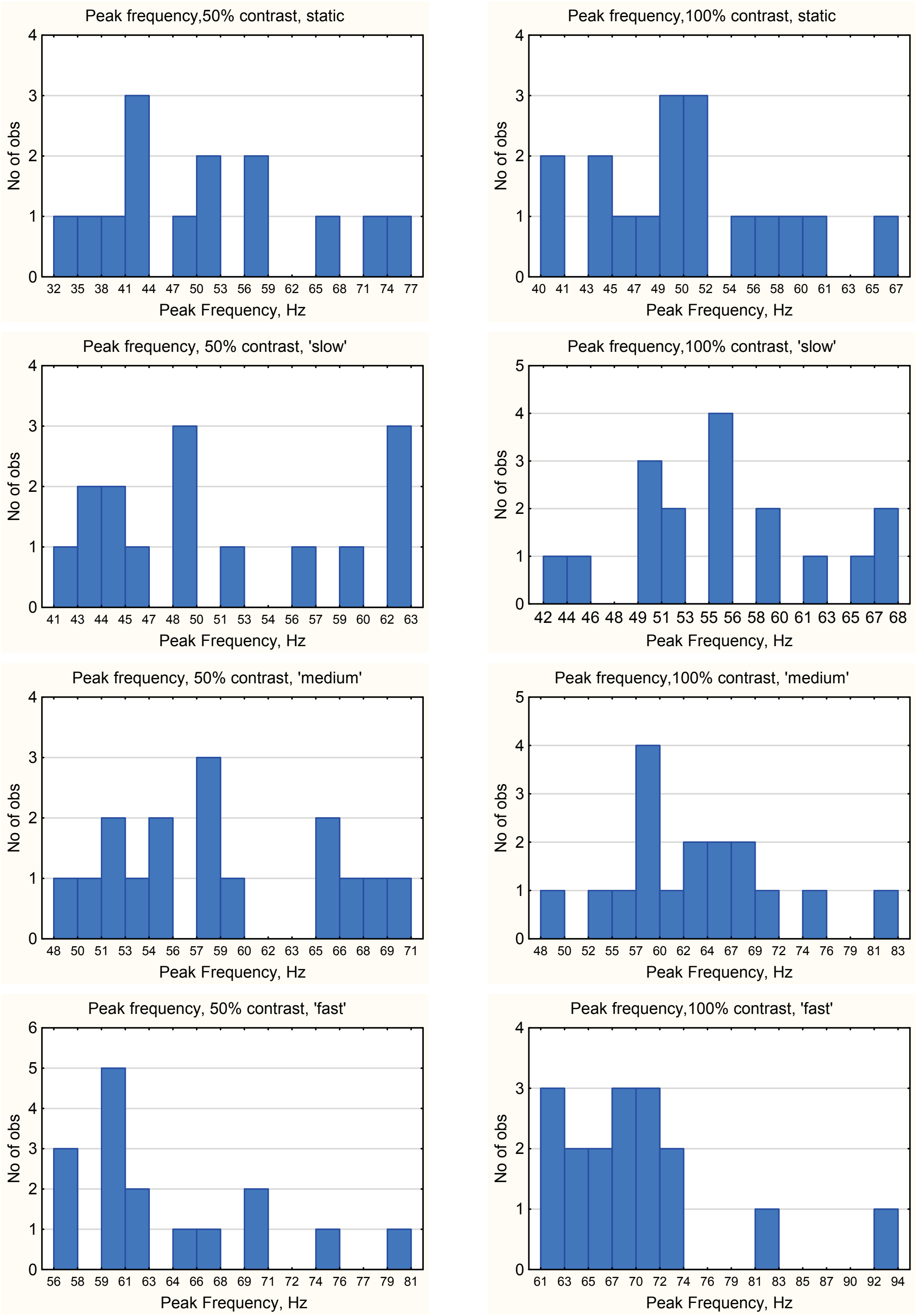


S2 Fig. Distributions of individual peak frequencies of gamma responses induced by visual gratins (static or moving at different velocities) at two lumunance contrasts.

*Orekhova et al. PLOS One, 2020*
