# Supplementary material for: Additive effect of contrast and velocity suggests the role of strong excitatory drive in suppression of visual gamma response
Source: PLoS One. 2020 Feb 13;15(2):e0228937. doi: 10.1371/journal.pone.0228937 (PMC7018047; doi:10.1371/journal.pone.0228937)
Supplement: S1 Table — Position of the voxel with maximal gamma response in the ‘static’, ‘slow’, ‘medium’, and ‘fast’ velocity conditions in each of the 17 participants. (PDF) [file pone.0228937.s005.pdf]

S1 Table A. 100% contrast: position of the voxel with maximal gamma response (GR) in the 'static', 'slow', 'medium', and 'fast' velocity conditions in each of the 17 participants. The results are presented for brain sources that met our two criteria: (1) a DICS-based source cluster with significant GR power increase was identified and (2) the GR power within the 25 voxel peak selection increased significantly (p<0.0001).

	AAL atlas anatomical label *				MNI coordinates of the voxel with maximal GR power (cm)											
Subj	Static	Slow	Medium	Fast	Static			Slow			Medium			Fast		
					X0	Y0	Z0	X 1	Y1	Z1	X2	Y2	Z2	X3	Y3	Z3
1	Calcarine_L	Calcarine_L	Calcarine_L	Calcarine_L	0.2	-10	0.2	0.2	-10	0.8	0.2	-10	0.8	0.2	-10	0.8
2	Calcarine_L	Calcarine_R	Calcarine_R	Calcarine_L	0.2	-8.8	0.8	0.8	-8.8	0.2	0.8	-8.8	0.2	0.2	-8.8	0.8
3	Calcarine_L	Occip_Sup_L	Calcarine_L	Calcarine_L	-0.4	-8.8	-1	-1	-9.4	0.8	-0.4	-8.8	0.8	-0.4	-8.8	0.8
4	Cuneus_R	Cuneus_R	Cuneus_R	Cuneus_L	2	-9.4	2	2	-9.4	0.8	2	-9.4	0.8	-0.4	-9.4	1.4
5	Cuneus_R	Cuneus_R	Cuneus_R	Cuneus_R	1.4	-10	1.4	1.4	-10	0.8	1.4	-10	0.8	1.4	-9.4	0.8
6	Calcarine_L	Calcarine_L	Calcarine_L	Calcarine_L	-0.4	-10	-0.4	-0.4	-10	0.2	-0.4	-9.4	0.2	0.2	-8.8	0.8
7	Calcarine_R	Calcarine_R	Calcarine_R	Calcarine_R	1.4	-8.8	0.8	0.8	-8.8	0.8	0.8	-8.8	0.8	0.8	-8.8	0.8
8	Cuneus_L	Occip_Sup_R	Cuneus_R	Occip_Sup R	-1	-9.4	2.6	2.6	-9.4	1.4	2	-9.4	1.4	2	-9.4	2
9	Calcarine_L	Calcarine_L	Calcarine_L	Calcarine_L	0.2	-9.4	0.2	0.2	-9.4	-0.4	0.2	-9.4	0.2	0.2	-9.4	-0.4
10	Occip_Mid_L	Occip_Mid_L	Occip_Mid_L	Occip_Mid_L	-1.6	-9.4	-1.6	-1.6	-9.4	0.2	-1.6	-9.4	0.2	-2.2	-10	0.8
11	Calcarine_L	Calcarine_R	Lingual_R	Lingual_L	-0.4	-8.8	0.8	0.8	-8.8	0.2	0.8	-8.8	-0.4	-0.4	-8.2	0.2
12	Calcarine_L	Calcarine_L	Calcarine_L	Calcarine_L	0.2	-10	0.2	0.2	-10	-0.4	0.2	-9.4	-0.4	-0.4	-9.4	0.2
13	Occip_Sup_L	Cuneus_R	Occip_Sup_L	Occip_Sup_L	-1.6	-9.4	1.4	1.4	-10	0.8	-1	-9.4	0.8	-1.6	-9.4	1.4
14	Calcarine_L	Calcarine_R	Calcarine_R	Calcarine_R	-0.4	-9.4	1.4	1.4	-8.8	1.4	0.8	-8.8	0.8	1.4	-8.8	1.4
15	Cuneus_L	Cuneus_L	Cuneus_L	Cuneus_L	-0.4	-9.4	-0.4	-0.4	-9.4	2	0.2	-9.4	2	0.2	-9.4	1.4
16	Calcarine_L[#]	Calcarine_L	Calcarine_L	Calcarine_L	-0.4	-9.4	0.2	0.2	-9.4	-1	-0.4	-9.4	-0.4	-0.4	-9.4	-0.4
17	Occip_Mid_L	Occip_Mid_L	Occip_Mid_L	Calcarine_L	-1	-10	-1	-1	-10	0.2	-1	-10	0.2	-0.4	-9.4	0.8

* According to the AAL atlas (N. Tzourio-Mazoyer, B. Landeau, D. Papathanassiou, F. Crivello, O. Etard, N. Delcroix, B. Mazoyer, and M. Joliot. *Automated Anatomical Labeling of Activations in SPM Using a Macroscopic Anatomical Parcellation of the MNI MRI Single-Subject Brain.* NeuroImage 2002. 15:273-289.)

[#] The maximal GR power increase occurred outside visual cortical areas; the position, however, was assessed for the voxel with maximal gamma increase within visual cortical areas.

S1 Table B. 50% contrast: position of the voxel with maximal gamma response (GR) in the 'static', 'slow', 'medium', and 'fast' velocity conditions in each of the 17 participants. The results are presented for brain sources that met our two criteria: (1) a DICS-based source cluster with significant GR power increase was identified and (2) the GR power within the 25 voxel peak selection increased significantly (p<0.0001).

	AAL atlas anatomical label *				MNI coordinates of the voxel with maximal GR power (cm)											
Subj	Static	Slow	Medium	Fast	Static			Slow			Medium			Fast		
					X0	Y0	Z0	X 1	Y1	Z1	X2	Y2	Z2	X3	Y3	Z3
1	Calcarine_L	Calcarine_L	Calcarine_L	Calcarine_L	0.2	-10	0.8	0.2	-10	0.8	0.2	-10	0.8	0.2	-10	0.8
2	Calcarine_L	Calcarine_L	Calcarine_L	Calcarine_L	-0.4	-9.4	0.2	0.2	-8.8	0.2	-0.4	-8.8	0.8	-0.4	-8.8	0.8
3	Cuneus_R[#]	Calcarine_L	Calcarine_L	Calcarine_L	2	-8.8	1.4	-0.4	-8.8	0.8	-0.4	-8.8	0.2	-0.4	-8.8	0.2
4	Cuneus_L	Cuneus_R	Cuneus_R	Cuneus_L	0.2	-9.4	1.4	2	-9.4	1.4	2	-9.4	0.8	0.2	-9.4	2
5	Cuneus_R	Cuneus_R	Cuneus_R	Cuneus_R	1.4	-9.4	0.8	1.4	-10	0.8	1.4	-9.4	0.8	1.4	-10	1.4
6	Calcarine_L	Calcarine_L	Calcarine_L	Calcarine_R	-0.4	-10	0.8	-0.4	-10	0.8	-0.4	-9.4	0.2	0.8	-9.4	0.8
7																
8		Cuneus_R	Occip_Sup_R					2	-9.4	1.4	2.6	-9.4	1.4			
9	Calcarine_L	Calcarine_L	Calcarine_L	Calcarine_L	0.2	-9.4	-0.4	0.2	-9.4	-0.4	0.2	-9.4	0.2	0.2	-9.4	0.2
10	Calcarine_R	Occil_Mid_L	Occip_Mid_L	Occip_Mid_L	0.8	-9.4	0.8	-1.6	-9.4	0.2	-1.6	-9.4	0.2	-2.2	-10	0.8
11	Lingual_L	Lingual_R	Calcarine_L	Calcarine_R	-1	-8.2	-0.4	0.8	-8.8	-0.4	-1	-8.8	0.2	1.4	-9.4	-0.4
12	Occip_Mid_L	Calcarine_L	Calcarine_L	Occip_Sup_L	-1	-10	0.2	-0.4	-9.4	0.2	-0.4	-9.4	0.2	-1	-9.4	0.2
13	Cuneus_L	Occip_Sup_L	Cuneus_L	Cuneus_L	0.2	-10	1.4	-1.6	-10	1.4	0.2	-10	1.4	0.2	-10	1.4
14	Calcarine_L	Calcarine_L	Lingual_R	Calcarine_L	-0.4	-10	0.2	-0.4	-9.4	-0.4	0.8	-8.8	-0.4	-0.4	-9.4	0.2
15	Cuneus_L	Cuneus_L	Cuneus_L	Occip_Sup_L	-0.4	-9.4	2	-0.4	-9.4	2	-0.4	-9.4	2	-0.4	-10	2
16		Calcarine_L	Calcarine_L	Calcarine_L				-0.4	-9.4	-0.4	-1	-9.4	-1	0.2	-9.4	-1
17	Occip_Sup_L	Occip_Mid_L	Occip_Mid_L	Cuneus_L	-1	-10	1.4	-1	-10	0.2	-1	-10	0.2	-0.4	-9.4	1.4

* According to the AAL atlas (N. Tzourio-Mazoyer, B. Landeau, D. Papathanassiou, F. Crivello, O. Etard, N. Delcroix, B. Mazoyer, and M. Joliot. *Automated Anatomical Labeling of Activations in SPM Using a Macroscopic Anatomical Parcellation of the MNI MRI Single-Subject Brain.* NeuroImage 2002. 15:273-289.)

[#] The maximal GR power increase occurred outside visual cortical areas; the position, however, was assessed for the voxel with maximal gamma increase within visual cortical areas.
