# Supplementary material for: Additive effect of contrast and velocity suggests the role of strong excitatory drive in suppression of visual gamma response
Source: PLoS One. 2020 Feb 13;15(2):e0228937. doi: 10.1371/journal.pone.0228937 (PMC7018047; doi:10.1371/journal.pone.0228937)
Supplement: S2 Table — Position of the voxel with maximal alpha-beta suppression in the ‘static’, ‘slow’, ‘medium’ and ‘fast’ velocity conditions in each of the 17 participants. (PDF) [file pone.0228937.s006.pdf]

S2 Table A. The 100% contrast: position of the voxel with maximal alpha-beta suppression in the 'static', 'slow', 'medium' and 'fast' velocity conditions in each of the 17 participants. The results are presented for brain sources that met our two criteria: (1) a DICS-based source cluster with significant alpha-beta suppression was identified and (2) the alpha-beta power within the 25 voxel peak selection significantly decreased during visual stimulation (p<0.0001).

	AAL atlas anatomical label *				MNI coordinates of the voxel with maximal decrease in power (cm)											
Subj	Static	Slow	Medium	Fast	Static			Slow			Medium			Fast		
					X0	Y0	Z0	X 1	Y1	Z1	X2	Y2	Z2	X3	Y3	Z3
1	Pariet_Sup_L	Pariet_Sup_L	Pariet_Sup_L	Occip_Mid_L	-2.8	-9.4	-0.4	-1	-10.6	0.2	-1.6	-10	0.8	-1	-10.6	0.2
2		Occip_Mid_R	Cuneus_R	Calcarine_R				2.6	-9.4	0.8	2	-8.8	0.8	2.6	-9.4	0.2
3	Occip_Mid_R	Occip_Inf_R	Occip_Inf_L	Occip_Mid_L	3.2	-9.4	0.2	4.4	-8.2	-1	-3.4	-8.8	-1	-3.4	-8.8	-0.4
4	Occip_Mid_R	Calcarine_L	Occip_Sup_R	Occip_Inf_R	3.2	-9.4	1.4	-1	-10	-0.4	2.6	-9.4	1.4	3.8	-9.4	-0.4
5	Calcarine_L	Lingual_L	Calcarine_L	Calcarine_L	0.2	-9.4	0.8	-1.6	-9.4	-1.6	-0.4	-10	0.8	-0.4	-10	0.8
6	Cuneus_R	Occip_Sup_R	Occip_Sup_L	Cuneus_L	1.4	-8.2	2	2	-8.2	2	-1.6	-9.4	1.4	-1	-9.4	1.4
7	Cerebelum R	Cerebelum R	Occip_Inf_L	Cerebelum R	-1.6	-10	-1.6	-1.6	-10	-1.6	-1.6	-9.4	-1	2.6	-9.4	0.8
8	Occip_Mid_R	Occip_Mid_L	Occip_Mid_R	Occip_Sup_L	4.4	-8.2	2	-2.8	-9.4	2	4.4	-8.2	2	-2.8	-9.4	2.6
9	Occip_Mid_R	Occip_Inf_R	Occip_Inf_R	Occip_Inf_L	3.8	-8.8	0.2	3.2	-8.2	-1.6	3.2	-8.8	-1.6	-2.2	-9.4	-0.4
10	Occip_Mid_R	Calcarine_L	Occip_Mid_R	Occip_Mid_R	2.6	-9.4	0.8	0.2	-10	0.2	3.8	-9.4	0.8	2.6	-9.4	0.8
11	Calcarine_R	Calcarine_R	Calcarine_R	Calcarine_R	1.4	-9.4	0.2	2	-9.4	0.2	1.4	-9.4	0.2	1.4	-9.4	0.2
12	Occip_Mid_L	Calcarine_R	Occip_Mid_L	Occip_Mid_L	-1.6	-10	0.8	2	-10	0.2	-2.2	-10	0.8	-1.6	-10	0.8
13	Occip_Mid_R	Calcarine_L	Occip_Mid_R	Occip_Mid_L	4.4	-8.2	2.6	-0.4	-10.6	-0.4	4.4	-8.2	2	-2.8	-9.4	1.4
14	Lingual_L	Occip_Sup_R	Lingual_L	Occip_Mid_R	-1.6	-10	-1.6	2.6	-9.4	1.4	-1.6	-10	-1.6	3.2	-9.4	1.4
15	Temp_Mid_R	Occip_Mid_R	Cuneus_R	Occip_Mid_R	4.4	-7.6	0.8	4.4	-7.6	1.4	2	-10	0.8	4.4	-7.6	0.8
16	Occip_Inf_L	Lingual_L	Lingual_L	Calcarine_L	-2.2	-8.8	-1	-1.6	-8.8	-1	-1.6	-8.8	-1	-1	-8.8	-0.4
17																

* According to the AAL atlas (N. Tzourio-Mazoyer, B. Landeau, D. Papathanassiou, F. Crivello, O. Etard, N. Delcroix, B. Mazoyer, and M. Joliot. *Automated Anatomical Labeling of Activations in SPM Using a Macroscopic Anatomical Parcellation of the MNI MRI Single-Subject Brain.* NeuroImage 2002. 15:273-289.)

S2 Table B. The 500% contrast: position of the voxel with maximal alpha-beta suppression in the 'static', 'slow', 'medium' and 'fast' velocity conditions in each of the 17 participants. The results are presented for brain sources that met our two criteria: (1) a DICS-based source cluster with significant alpha-beta suppression was identified and (2) the alpha-beta power within the 25 voxel peak selection significantly decreased during visual stimulation (p<0.0001).

	AAL atlas anatomical label *				MNI coordinates of the voxel with maximal decrease in power (cm)											
Subj	Static	Slow	Medium	Fast	Static			Slow			Medium			Fast		
					X0	Y0	Z0	X 1	Y1	Z1	X2	Y2	Z2	X3	Y3	Z3
1	Pariet_Sup_L	Pariet_Sup_L	Pariet_Sup_L	Occip_Mid_L	-2.8	-9.4	-0.4	-1	-10.6	0.2	-1.6	-10	0.8	-1	-10.6	0.2
2		Occip_Mid_R	Cuneus_R	Calcarine_R				2.6	-9.4	0.8	2	-8.8	0.8	2.6	-9.4	0.2
3	Occip_Mid_R		Occip_Inf_L	Occip_Mid_L	3.2	-9.4	0.2				-3.4	-8.8	-1	-3.4	-8.8	-0.4
4	Occip_Mid_R	Calcarine_L	Occip_Sup_R	Occip_Inf_R	3.2	-9.4	1.4	-1	-10	-0.4	2.6	-9.4	1.4	3.8	-9.4	-0.4
5	Calcarine_L	Lingual_L	Calcarine_L	Calcarine_L	0.2	-9.4	0.8	-1.6	-9.4	-1.6	-0.4	-10	0.8	-0.4	-10	0.8
6	Cuneus_R	Occip_Sup_R	Occip_Sup_L	Cuneus_L	1.4	-8.2	2	2	-8.2	2	-1.6	-9.4	1.4	-1	-9.4	1.4
7	Cerebelum R	Cerebelum R	Occip_Inf_L	Cerebelum R	-1.6	-10	-1.6	-1.6	-10	-1.6	-1.6	-9.4	-1	2.6	-9.4	0.8
8	Occip_Mid_R	Occip_Mid_L	Occip_Mid_R	Occip_Sup_L	4.4	-8.2	2	-2.8	-9.4	2	4.4	-8.2	2	-2.8	-9.4	2.6
9	Occip_Mid_R	Occip_Inf_R	Occip_Inf_R	Occip_Inf_L	3.8	-8.8	0.2	3.2	-8.2	-1.6	3.2	-8.8	-1.6	-2.2	-9.4	-0.4
10	Occip_Mid_R	Calcarine_L	Occip_Mid_R	Occip_Mid_R	2.6	-9.4	0.8	0.2	-10	0.2	3.8	-9.4	0.8	2.6	-9.4	0.8
11	Calcarine_R	Calcarine_R	Calcarine_R	Calcarine_R	1.4	-9.4	0.2	2	-9.4	0.2	1.4	-9.4	0.2	1.4	-9.4	0.2
12		Calcarine_R	Occip_Mid_L	Occip_Mid_L				2	-10	0.2	-2.2	-10	0.8	-1.6	-10	0.8
13	Occip_Mid_R	Calcarine_L	Occip_Mid_R	Occip_Mid_L	4.4	-8.2	2.6	-0.4	-10.6	-0.4	4.4	-8.2	2	-2.8	-9.4	1.4
14	Lingual_L	Occip_Sup_R	Lingual_L	Occip_Mid_R	-1.6	-10	-1.6	2.6	-9.4	1.4	-1.6	-10	-1.6	3.2	-9.4	1.4
15	Temp_Mid_R	Occip_Mid_R	Cuneus_R	Occip_Mid_R	4.4	-7.6	0.8	4.4	-7.6	1.4	2	-10	0.8	4.4	-7.6	0.8
16	Occip_Inf_L	Lingual_L	Lingual_L	Calcarine_L	-2.2	-8.8	-1	-1.6	-8.8	-1	-1.6	-8.8	-1	-1	-8.8	-0.4
17	Occipi_Inf_R	Calcarine_R	Occip_Sup_R	Calcarine_R	2	-10	0.2	3.8	-9.4	-1	-1.6	-10	0.8	2	-10	0.8

* According to the AAL atlas (N. Tzourio-Mazoyer, B. Landeau, D. Papathanassiou, F. Crivello, O. Etard, N. Delcroix, B. Mazoyer, and M. Joliot. *Automated Anatomical Labeling of Activations in SPM Using a Macroscopic Anatomical Parcellation of the MNI MRI Single-Subject Brain.* NeuroImage 2002. 15:273-289.)

S3 Table. Partial correlations between the peak frequencies of GRs elicited by visual gratings drifting with different velocities in the two contrast conditions. Age was taken as a nuisance variable. The frequency was evaluated only for reliable GRs (see Methods).

	100% contrast			
	0°/s	1.2°/s	3.6°/s	6.0°/s
0°/s		**0,83*** **	**0.65***	0.36
1.2°/s			**0.92*** **	**0.72** **
3.6°/s				**0.87*** **
	50% contrast			
	0°/s	1.2°/s	3.6°/s	6.0°/s
0°/s		**0.68** **	**0.6***	**0.58***
1.2°/s			**0.93*** **	**0.84*** **
3.6°/s				**0.91*** **

*p<0.05, **p<0.01, *** p<0.001
