# Supplementary material for: Additive effect of contrast and velocity suggests the role of strong excitatory drive in suppression of visual gamma response
Source: PLoS One. 2020 Feb 13;15(2):e0228937. doi: 10.1371/journal.pone.0228937 (PMC7018047; doi:10.1371/journal.pone.0228937)
Supplement: S3 Table — Partial correlations between the peak frequencies of GRs elicited by visual gratings drifting with different velocities in the two contrast conditions. (PDF) [file pone.0228937.s007.pdf]

S3 Table. Partial correlations between the peak frequencies of GRs elicited by visual gratings drifting with different velocities in the two contrast conditions. Age was taken as a nuisance variable. The frequency was evaluated only for reliable GRs (see Methods).

	100% contrast			
	0°/s	1.2°/s	3.6°/s	6.0°/s
0°/s		**0,83*** **	**0.65***	0.36
1.2°/s			**0.92*** **	**0.72** **
3.6°/s				**0.87*** **
	50% contrast			
	0°/s	1.2°/s	3.6°/s	6.0°/s
0°/s		**0.68** **	**0.6***	**0.58***
1.2°/s			**0.93*** **	**0.84*** **
3.6°/s				**0.91*** **

*p<0.05, **p<0.01, *** p<0.001
